# Evaluating zoonotic metacestodes: gross and histopathological alterations of beef in north–west Ethiopia one health approach for meat inspection and animal management

**DOI:** 10.3389/fvets.2024.1411272

**Published:** 2024-07-19

**Authors:** Dessie Alene, Moges Maru, Yitayew Demessie, Asnakew Mulaw

**Affiliations:** ^1^Department of Pathobiology, College of Veterinary Medicine and Animal Science University of Gondar, Gondor, Ethiopia; ^2^Department of Biomedical, College of Veterinary Medicine and Animal Science University of Gondar, Gondor, Ethiopia

**Keywords:** cattle, cyst, cysticercosis, histopathology, hydatidosis, zoonotic metacestode

## Abstract

Zoonotic metacestodes present a significant threat to both veterinary and public health. Specifically, the prevalence of metacestodes is often concentrated among consumers of raw meat and underdeveloped countries. The objective of this study was to estimate the prevalence of condemned red offal and examine the gross and histopathology features of zoonotic metacestodes. A cross-sectional study was conducted from November 2022 to July 2023 at the Bahir Dar municipal abattoir. A simple random sampling method employed in the abattoir survey to investigate pathological changes of offal and its rate of condemnation. Following a gross inspection of the red offal, representative tissue samples collected and preserved in 10% neutral buffered formalin. Subsequently, the size and number of cysts determined, and their viability and fertility evaluated. Hematoxylin and eosin staining utilized to analyze various lesions with microscope. A total of 340 cattle examined and 7.5% red offal condemned due to hydatid cysts 4.12% in the lungs, 3% in the liver, 0.6% in the kidneys, and 0.9% in other organs. Red offal condemned due to *Cysticercus bovis* 0.6% in the liver and 0.3% in the tongue. A statistically significance relationship was found between lung rejection due to hydatidosis (*p* < 0.05), body condition score, and origin of the animal. Among the detected calcified cysts, 83.34% of *C. bovis* and 47.62% of hydatid cysts. Histopathological examination revealed hydatid cysts and their oncospheres within the portal circulation, as well as necrotized, calcified daughter cysts observed on Bowman’s capsule. The alveoli and bronchiole parenchyma compressed with pressure of protoscolices and it infiltrated by eosinophils. The cyst wall is attached to the thick hepatic capsule of the liver, with the hepatic parenchyma displaying islands of irregular hepatocytes. *Cysticercus bovis* detected in the deteriorated and necrotized muscle bundles, along with granulomatous lesions characterized by infiltration of mononuclear cells. Gross and histological examinations is invaluable tool for diagnosing hydatidosis and cysticercosis, providing well-organized baseline data to enhance our understanding the burden of zoonotic metacestodes.

## Introduction

Ethiopia has the largest livestock population in Africa, with an estimated approximately 70 million cattle, 40 million sheep, 51 million goats, 8 million camels, and 63 million chickens (1). The majority of the country’s livestock population is found in the highlands, where mixed agriculture is practiced. It is the backbone of the country and serves as power for drought, meat, milk, and monetary income (2).

Rural livestock systems faced challenges such as conventional management systems, lack of ration nutrition, inadequate disease control strategy and veterinary services not addressed well. However, livestock in Ethiopia has a huge livelihood impact on the society as a food security and economic source. The tremendous losses brought by helminth parasites also known as “the silent predators” are intolerable (3).

Annually huge numbers of cattle are slaughtered in Ethiopia for human consumption. Organs and carcasses were condemned due to different infectious diseases and lesions at the abattoir. The condemnation of red offal result in significant financial losses in the meat industry. The meat business in the developing country has a loss from parasites than from any other disease. Fasciolosis, hydatidosis, and cysticercosis are common parasite infections that afflict the liver, lungs, kidneys, spleens, hearts, tongues, and other organs (4).

Cysticercosis and hydatidosis are caused by cestode parasites and the infection is acquired after ingestion of the parasite’s egg which contains an infective oncosphere. In the intermediate host, the parasite develops into an infective metacestode. *Echinococcus granulosus* is a common aetiological agent of echinococcosis (CE). It has a worldwide distribution, particularly in countries where pastoral activities are prominent (5).

Many herbivorous species are susceptible to *E. granulosus* parasites. Parasites of the genus *Taenia*, which cause cysticercosis, are typically very specific for both intermediate and definitive host species. *T. saginata* are an economically important species causing cysticercosis in cattle. Humans act as definitive hosts for *T. saginata* (6). *Cysticercus bovis* cysts are frequently found in the kidney, heart, liver, oesophagus, diaphragm, tongue, and masticatory muscles in intermediate hosts. Bovine cysticercosis usually does not result in clinical disease in cattle; therefore, diagnosis is based on postmortem examinations (7).

Cystic hydatidosis and cysticercosis are parasitic foodborne zoonosis diseases which affect both animals and humans. The practice of backyard animal slaughter, coupled with the absence of stringent meat inspection standards and the feeding of condemned offal to dogs, contributes to the contamination of pastures and grazing lands. This makes it easier to maintain the *E. granulosus* life cycle, cystic hydatidosis, as a result, the high rate of infection of susceptible host (8). Taeniasis in humans and cysticercosis in cattle are frequently reported in Ethiopia (9).

The presence of cysticerci and hydatid cysts was determined by visual inspection and palpation, followed by many incisions in the liver, kidney, lung, kidney, heart, and tongue (10). Hydatid cysts found in the liver, lung, and heart. The cysts filled with a clear hydatid fluid and had an exterior fibrous layer covering the inner germinal layer. The findings confirmed that the germinal layer is where parasite growth occurs most frequently (11).

Histopathological examination revealed hepatocyte atrophy and necrosis, and collapsed lung tissue adjacent to the cyst wall. All other sections showed fibrous tissue (capsule) and a cellular reaction. Most of the tissue the sections had cystic layers (laminated and germinative layers). Monocytes were the most prevalent form of inflammatory cells was seen. Cellular infiltrations were generally diffuse, and inflammatory cells mainly consisted of mononuclear cells and neutrophils infiltrate (12).

*Taenia saginata* cysticercosis diagnosis can be easily made by gross examination and using a stereoscopic microscope, if the cysticerci are viable, or if an intact or partial metacestode can be seen in a recently degenerated cyst. Cysts can be viewed grossly as early as 11 days post-infection, at which time they are about 2.5 mm in diameter. The inspection protocol involves incision and palpation of the tongue, internal and external masseters, oesophagus, heart, and diaphragm (13). Histologically, the majority of lesions included a mass of necrotic cellular debris in the center, which occasionally contained calcareous corpuscles or other parasite remains, and a granulomatous cellular zone that was primarily made up of lymphocytes, plasma cells, and eosinophils. At the border between the cellular zone and the necrotic core, giant cells were frequently observed. Collagen, fibrocytes, and fibroblasts came together to form a unique capsule around the cellular zone (14). Metacestode parasites are one of the neglected tropical diseases that affect humans, canines, and livestock. Thus, the objective of this study was to identify and describe both macroscopically and microscopically, the lesion on condemned red offals related to the zoonotic metacestode parasites that slaughter cattle at the municipal abattoir in Bahir Dar.

## Materials and methods

### Study area

The study was conducted in the Bahir Dar municipal abattoir from November 2022 to July 2023. The capital city of the Amhara National Regional State is Bahir Dar Town, which is situated 565 km to the north–west of Addis Abeba at 11°35′37.10″N latitude and 37°23′26.77″E longitude on the south of Lake Tana. The upper watershed of the Blue Nile River has an elevation range of 1,500–1,820 m above sea level and receives 1,500 mm of rainfall annually on average. The average yearly temperature is 23°C, and the climate is influenced by Lake Tana and the River Abay. The region practices mixed farming, producing both crops and livestock (15).

### Study population

The Amhara Regional State is geographically centered in Bahir Dar, where a sizable population of cattle is transported for the live markets. Animals for slaughtering come primarily from Bahir Dar and the surrounding district. The indigenous breed of animals and the semi-intensive management system are the ones headed for slaughtering. Every cattle that enters the abattoir appears to be male, apparently healthy, and in a variety of age and body condition categories.

At the survey, potential risk factors were documented and evaluated such as interest, origin, age, and body conditions status of the animals. The age of cattle was determined based on the dentition formula adopted by Parish and Karisch (16) and was categorized into ≤5 years young and >5 years adults. Additionally, Nicholson and Butterworth’s (17) criteria were used to evaluate the body condition score of cattle, classifying the cattle as poor, medium, or good. Catlle that are deemed unfit for slaughter due to their clinical illness and emaciation were removed during the antemortem inspection. During the study period, 18–25 cattle had been slaughtered at the municipal abattoir in Bahir Dar in a week.

### Sample size determination

The sample size was determined using a formula adopted by (18) at 5% absolute prevalence and a 95% confidence interval, considering the previous prevalence (33.05%) (17) of bovine zoonotic metacestodes. *n* = (1.96)^2^ Pexp (1-Pexp)/*d*^2^.

Where, *n* = sample size; 1.96 = multiplier of the 95% confidence interval; Pexp = expected prevalence 33.05% and *d*^2^ = desired absolute precision (5%), *n* = (1.96)^2^ 0.3305 (1–0.3305)/(0.05)^2^ = 340 (minimum sample size for this study) (0.05)^2^.

### Sampling method

After the study area, Bahir Dar was selected purposively, and samples were collected with random sampling in the abattoir. The red offals collected those which have gross cystic lesions at postmortem inspection. Those positive for the cysts were assigned histopathological examination and cyst characterization. However, the organs free from any visible gross lesions were excluded from the sampling.

### Post mortem inspection

An active abattoir survey was conducted by employing routine meat inspection procedures AM (antemortum meat) and PM (postmortem meat) (17) on 340 cattle slaughtered. During inspections antemortem and postmortem, risk factors recorded with serious identification numbers for each cattle. After slaughtering, the same identification numbers gave for all red offals for each slaughtering cattle. The predilection organs of each zoonotic metacestode thoroughly examined. Thorough visual inspection, palpation, and systematic incision of each visceral organ carried out during PM. Cysticercosis assessed with a deep, linear incision from the base to athe apex part of tongue. The heart was incised from its base to its apex to open the pericardium, and an incision was also made in the cardiac muscle for a detailed examination. An examination of the kidneys, liver, lungs, and spleen was also conducted for hydatidosis according to PM method. The samples, cysticerci/haydatid cysts-infected cattle collected at PM and taken to the Bahir Dar Regional Parasitology Laboratory. After that, the size and number of cysts were determined. The cyst’s viability and fertility were evaluated. The size of a hydatid cyst was measured and classified according to its size such as small when it <4 cm, medium when the diameter <8 and >4 cm, and large when the diameter >8 cm, according to (16). For histopathology diagnosis, the lung, liver, kidney, and tongue with gross lesions were collected on size 1 cm^3^ 10% neutral buffered formalin (17) and taken to the University of Gondar Veterinary Pathology Laboratory.

### Sample processing

#### Parasitological examination

*Cysticercus bovis* were incubated for 1–2 h at 37°C using a 40% ox bile solution diluted in normal saline. Next, the scolex was examined under a microscope by being sandwiched between two glass slides. The cysts are categorized as viable if the scolex evaginates during the incubation period, as per the description provided by Megersa et al. (19).

Before each hydatid cyst was placed in a sterile container and examined under a 40× microscope for protoscoleces, it was grossly inspected for any evidence of calcification. The vitality of protoscolices was assessed using the motion of the flame cells and staining with a 0.1% aqueous solution of eosin, as described (17). Protoscolices were deemed viable if they possessed germinal membranes; in the absence of protoscolices, the sample was deemed non-fertile. Viable protoscolices did not absorb the dye.

#### Histopathological examination

Following appropriate fixation, samples of tissues with cystic lesion types from the liver, kidney, and tongue have been sliced, dehydrated in ethyl alcohol in increasing grades, cleaned in xylene, impregnated with melted paraffin wax, and embedded. Tissue slices, about 4–5 μm thick, were cut and stained using hematoxylin and eosin (H & E) by protocol (17, 20, 21). Under a light microscope with low to high powers of magnification (4–40×), stained slides were mounted using Distrene plasticizer/Dibutyl phthalate xylene (DPX), and photomicrographs were then taken for documentation.

### Data management and analysis

Data obtained from the abattoir were recorded, stored in Microsoft Excel, and transferred to STATA version 14 for the descriptive analysis of frequency and percentages of the rejection rate of red offals results. Associations between outcome and explanatory variables for all units of analysis were investigated using Pearson’s chi-square (*χ*^2^). For all the analyses, the confidence level (CL) is at 95%, and *p* < 0.05 was set for significance.

## Results

### Condemned red offal

Inspection of 340 cattle organs revealed that 4.12% of the lungs, 3% of livers, and 0.6% of kidneys, together with 76.5 percent of the red offals, were afflicted with hydatidosis illness and were therefore deemed unfit for human consumption. As shown in Table 1, cysticercosis led to the rejection of livers (0.6%), tongues (0.3%), and whole red offal (0.9%) as unfit for human consumption.

**Table 1:** Rate of red offals condemnation associated with zoonotic metacestodes at Bahir Dar municipal abattoir.

Organs	No. examined organ (*n*)	Hydatid cysts	%	Cysticercosis	%	Total	%
Lung	340	14	4.12	0		14	4.12
Liver	340	10	3	2	0.6	12	3.53
Heart	340	0		0		0	
Kidney	340	2	0.6	0		2	0.6
Tongue	340	0		1	0.3	1	0.3
Spleen	340	0		0		0	
Total		26	7.65	3	0.9	29	8.53

No. in the table indicate number examined organs.

In cattle’s’ of various ages, the rejection rate of red offal due to zoonotic metacestode investigated As on the young animals cattle reject rate was their liver (3.57%) and lung (3.57%). Moreover, the adult’s (4.5%) lung, (2.5%) liver, and (1%) kidney rejected, as shown in Table 2 the rejection of the tongue in young cattle (0.71%) and the liver in the adult cattle (1%) by endorsed with cysticercosis.

**Table 2:** Risk factors of red offals condemnation from cattle slaughtered at Bahir Dar municipal abattoir infected by hydatidosis.

Risk factor		No.	Lung	%	Liver	%	Kidney	%
Age	Young	140	5	3.57	5	3.57	–	
	Adult	200	9	4.5	5	2.5	2	1
	*X*^2^ (*p*-value)		0.1799	0.671	0.3312	0.565	1.4083	0.235
Body condition score	Poor	75	8	10.67	3	4	2	2.67
	Medium	180	4	2.22	5	2.78	–	
	Good	85	2	2.35	2	2.35	–	
	*X*^2^ (*p*-value)		10.4559	0.005	0.4144	0.813	7.1085	0.029
Animal origin	Bahidar Zuria	78	2	2.56	3	3.85	1	1.28
	Adet	150	7	4.67	2	1.33	–	
	Este	73	–	12.82	4	5.48	1	1.37
	Debretabor	39	5		1	2.56	–	
	*X*^2^ (*p*-value)		11.2080	0.011	6.8674	0.076	2.5231	0.471
Total		340	14	4.12	10	2.94	2	0.6

No. in the table indicate number examined organs.

In terms of animal body condition scores, 10.67% of lungs, 4% of livers, and 2.67% of kidneys rejected from the poor body condition scores of cattle. Additionally, 2.22% of lungs and 2.78% of livers rejected from medium body condition scores cattle. Among cattle with good body condition scores, 2.35% of lungs and 2.35% of livers rejected due to Hydatid cysts, as indicated in Table 2.

The tongue the of cattle condemned such as 1.33% of poor body condition score, the liver 0.56% from medium body condition scores, and the liver 1.18% from those with good body condition scores were rejected due to cysticercosis, as shown in Table 3. Animals from different origins had varying rates of lung 2.56%, liver 3.85%, and kidney 1.28% found in Bahirdar Zuria, 4.67% from Adet, and 1.33% from Este 5.48%.

**Table 3:** Association of risk factors with red offal condemnation rate of cattle slaughtered at Bahir Dar municipal abattoir infected by *Cysticercus bovis*.

Risk factor			Tongue		Liver	
		No.	No.	%	No.	%
Age	Young	140	1	0.71		
	Adult	200			2	1
	*X*^2^ (*p*-value)		1.4328	0.231	1.4083	0.235
Body condition score	Poor	75	1	1.33		
	Medium	180			1	0.56
	Good	85			1	1.18
	*X*^2^ (*p*-value)		3.5438	0.170	0.9500	0.622
Origin	Birdar zuria	78			1	1.82
	Adet	150	1	0.67		
	Este	73				
	Debretabor	39			1	2.56
	*X*^2^ (*p*-value)		1.2704	0.736	4.5653	0.207
Total		340	1		2	

No. in the table indicate number examined organs.

The red offals condemned because of hydatidosis 1.82% liver from Bahirdar zuria (0.67%), Adet and 2.56% Debretabor shown in Table 3. Lung condemned rate 12.82% due to cysticercosis. There were statistically significance relationships between kidney rejection rate and body condition score of the cattle (*χ*^2^ = 7.1085, *p* = 0.029) and origin (*χ*^2^ = 11.2080, *p* = 0.011), as well as between lung rejection rate and body condition score (*χ*^2^ = 10.4559, *p* = 0.005).

### Cysts characteristics

From the cysts count, 84 hydatid cysts 17.86% fertile and contained protoscolices, whereas the remaining 82.14% hydatid cysts infertile. Also, 5.95% of them were viable, 11.9% were non-viable cysts, 34.52% were sterile, and 47.62% were calcified cysts, as indicated in Table 4.

**Table 4:** Fertility and viability status of hydatidosis disease in different condemned red offal.

Organ	Total cyst		Viable		Non-calcified cysts					
					Non-viable		Sterile cysts		Calcified cysts	
	No.	%	No.	%	No.	%	No.	%	No.	%
Liver	23	27.38	2	8.7	3	13.04	8	34.78	10	43.48
Lung	56	66.67	1	1.79	5	8.93	21	37.5	29	51.79
Kidney	5	5.95	2	40	2	40	–	–	1	20
Total	84	100	5	5.95	10	11.9	29	34.52	40	47.62

No. in the table indicate number examined organs.

The viability of the *C. bovis* cysts was assessed, and from the total, 12 cysts (16.67%) were viable and 83.34% were calcified cysts, as indicated in Tables 5, 6.

**Table 5:** Viability status of *Cysticercus bovis* in different condemned red offals.

No. of non-calcified cyst								
Organs inspected	No. of positive	Total cyst counted		Viable		Non-viable	Calcified	
		No.	%	No.	%	No.	No.	%
Tongue	1	7	58.34	–		–	7	100
Liver	2	5	41.67	2	40	–	3	60
Total	3	12	100	2	16.67	–	10	83.34

No. in the table indicate number based on the size of the cysts, 59.1% were small, 27.27% were medium, and the rest 13.63% were large as indicated in Table 6.

**Table 6:** The size of the hydatid cysts in the condemned red offal.

Offal		Small size cysts		Medium size cysts		Large size cysts	
Organs	No. cysts	No. cysts	%	No. cysts	%	No. cysts	%
Liver	13	8	61.54	4	30.77	1	7.69
Lung	27	16	59.26	7	25.96	4	14.81
Kidney	4	2	50.0	1	25.0	1	25.0
Total	44	26	59.1	12	27.27	6	13.63

No. cysts in the table indicate number cysts examined on the organs.

### Hydatid cysts of lung

**Figure 1:**
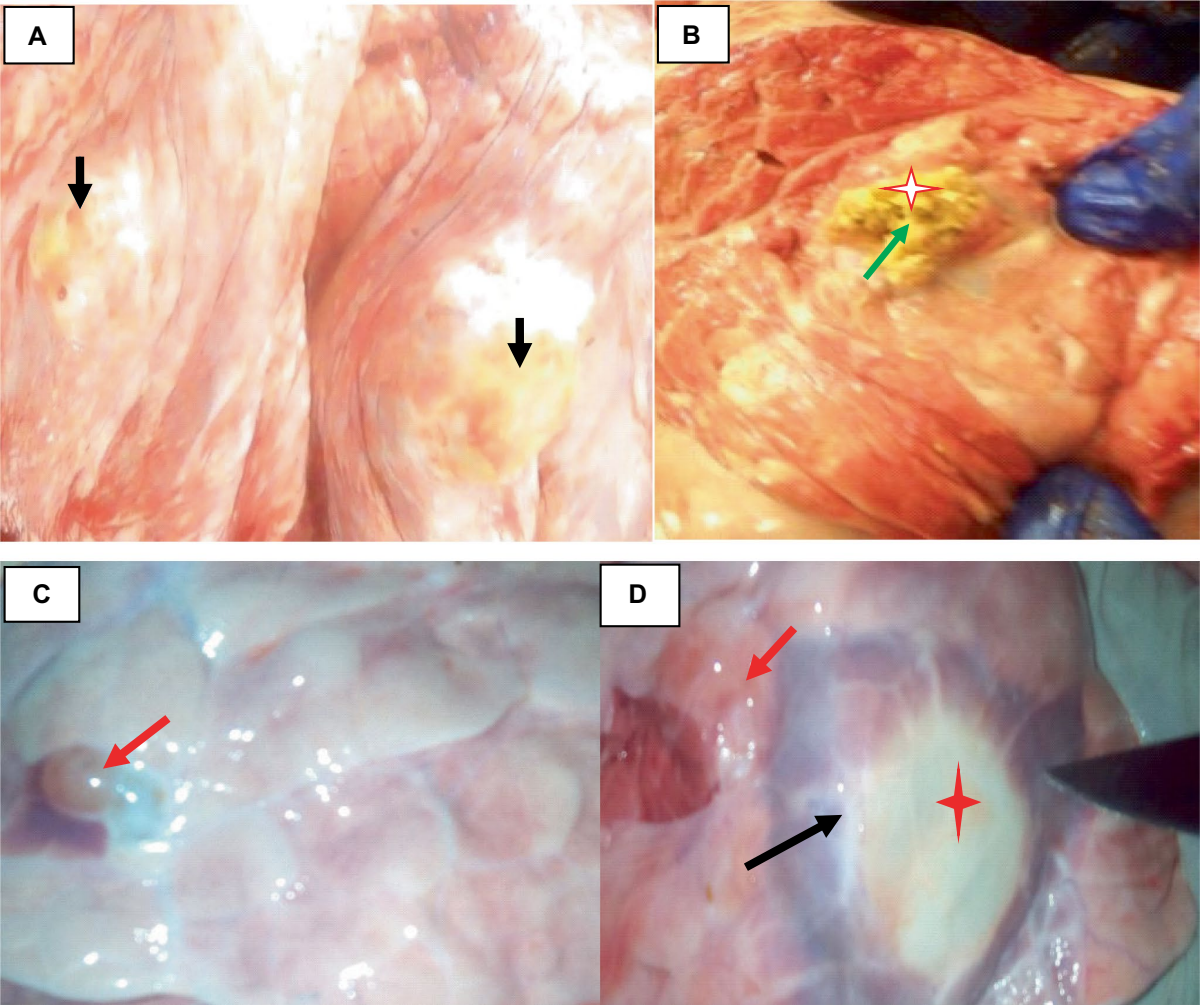
Hydatidosis in cattle lung. **(A)** The emphysematous and pale cysts are present in the left and right lung lobes (black arrows). **(B)** Depositions of calcium salt (green arrows). **(C)** Indicates a congested lung with cysts. **(D)** Edematous lung (black arrows) and cysts embedded (star).

The hydatidosis, rejection rate for cystic lung 4.12% and from the total condemned lung two lungs have double hydatid cysts. One lung has uniform in size cysts and the other one lung has variable size of cysts. Observing the lung closely, it appeared heavy, atelectatic or emphysema and pale. The cysts have different sizes and location of on the lung surface. The lung parenchyma was found to include cysts of varying sizes, which resulted in the lung’s substantial growth and weight increase. As said, the cysts appeared gray or yellowish and contained clear turbid fluids as indicated in Figure 1.

**Figure 2:**
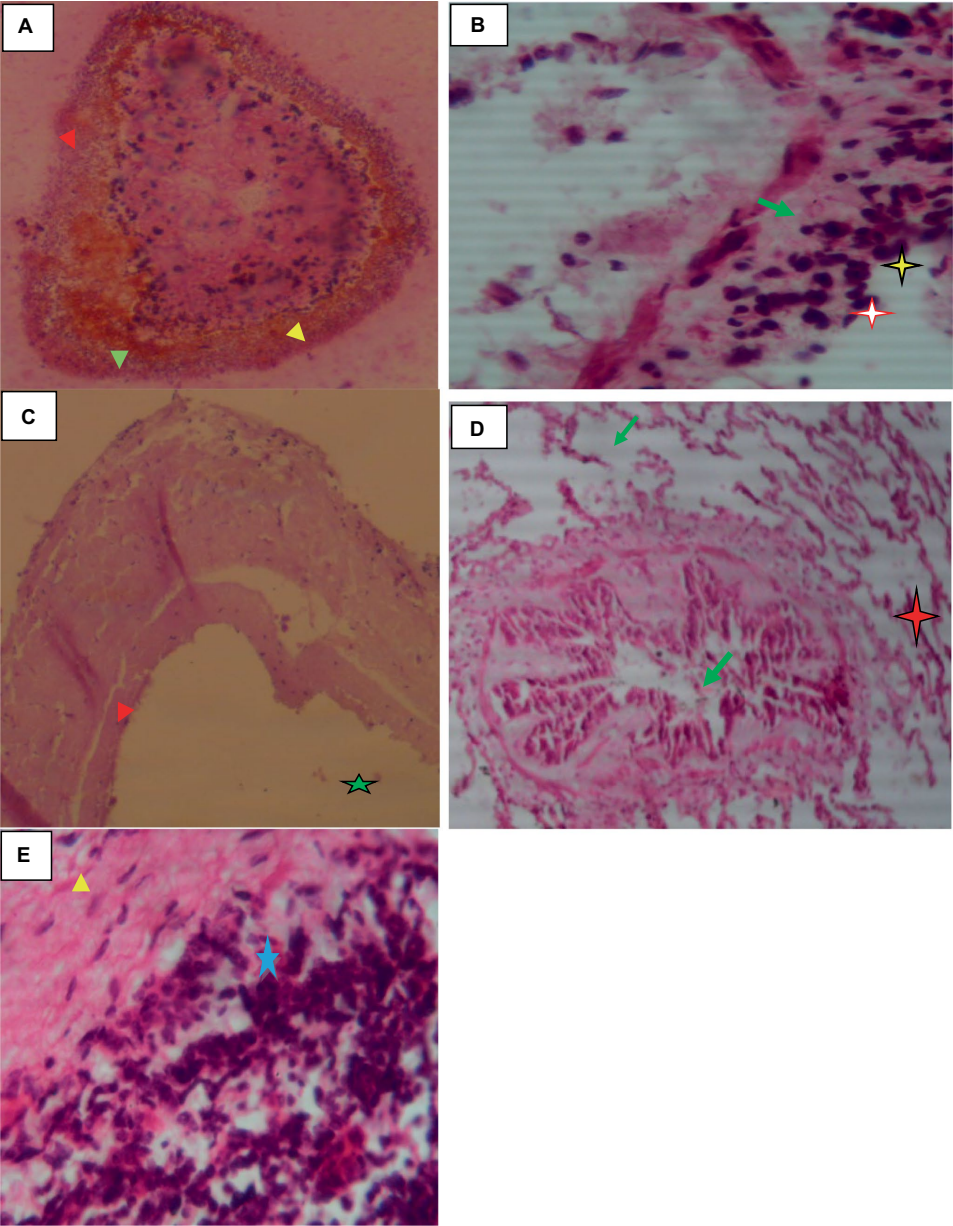
Hydatidosis in cattle lung 40× magnifications. **(A)** Calcified daughter cyst with three layers. **(B)** Remnant of the laminated layer (red arrows) and some mononuclear infiltration (star). **(C)** Laminated layer (red arrowhead) and germinative layer (star). **(D)** Compressed alveoli (star), bronchiolar parenchyma with stenosis and fibrosis (green arrows). **(E)** Mononuclear and plasma cell infiltration (star).

Histologically, the hydatid cysts consist of up of three layers: the laminated middle ectocyst layer, the fibrous outer pericyst, and the germinative inner endocyst layer, which is made up of daughter cysts. Macrophages, lymphocytes, and eosinophils had infiltrated the region, and the lung parenchyma surrounding the cyst was fibrotic and edematous. Additionally, calcified material had accumulated on the periphery as shown in Figure 2.

### Hydatid cysts liver

**Figure 3:**
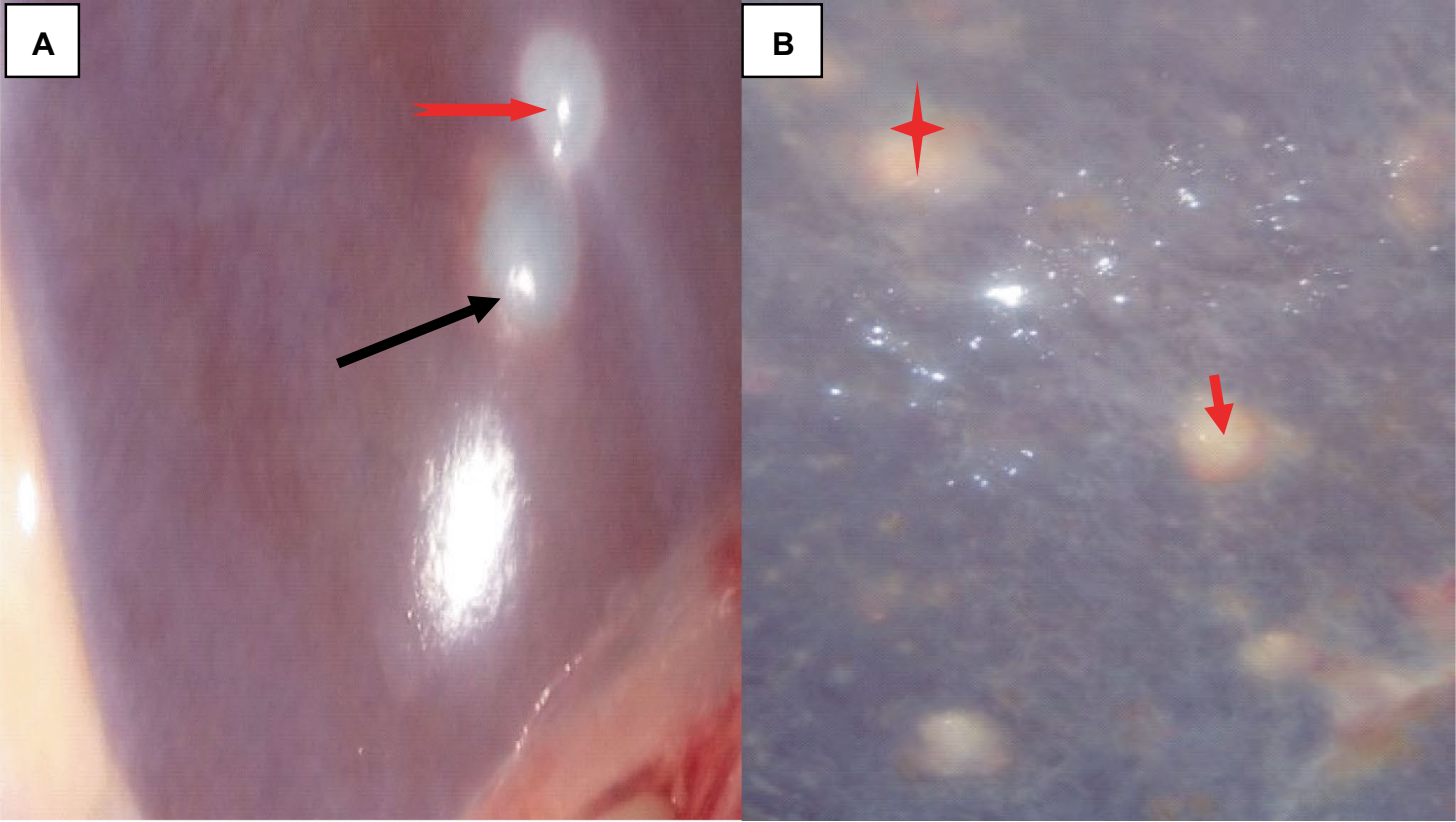
Cattle hepatic hydatidosis. **(A)** Liver with a localized cystic surface (red arrow) and a blunt edge (black arrow). **(B)** Liver with a calcified area (star) and a generalized cystic surface (arrow).

The percentage of hydatidosis-related condemnation of liver organ from the slaughtered animal 3%. Two livers have double cysts both cysts were medium size. It loaded with several cysts of different sizes filled with clear fluid. At the site of the incision, the cysts cavities with varying diameters and capsule thicknesses. Certain cysts had thin capsules that were white to slightly yellowish, while others had thick, fibrous capsules that were yellowish and included calcified material as shown in Figure 3.

**Figure 4:**
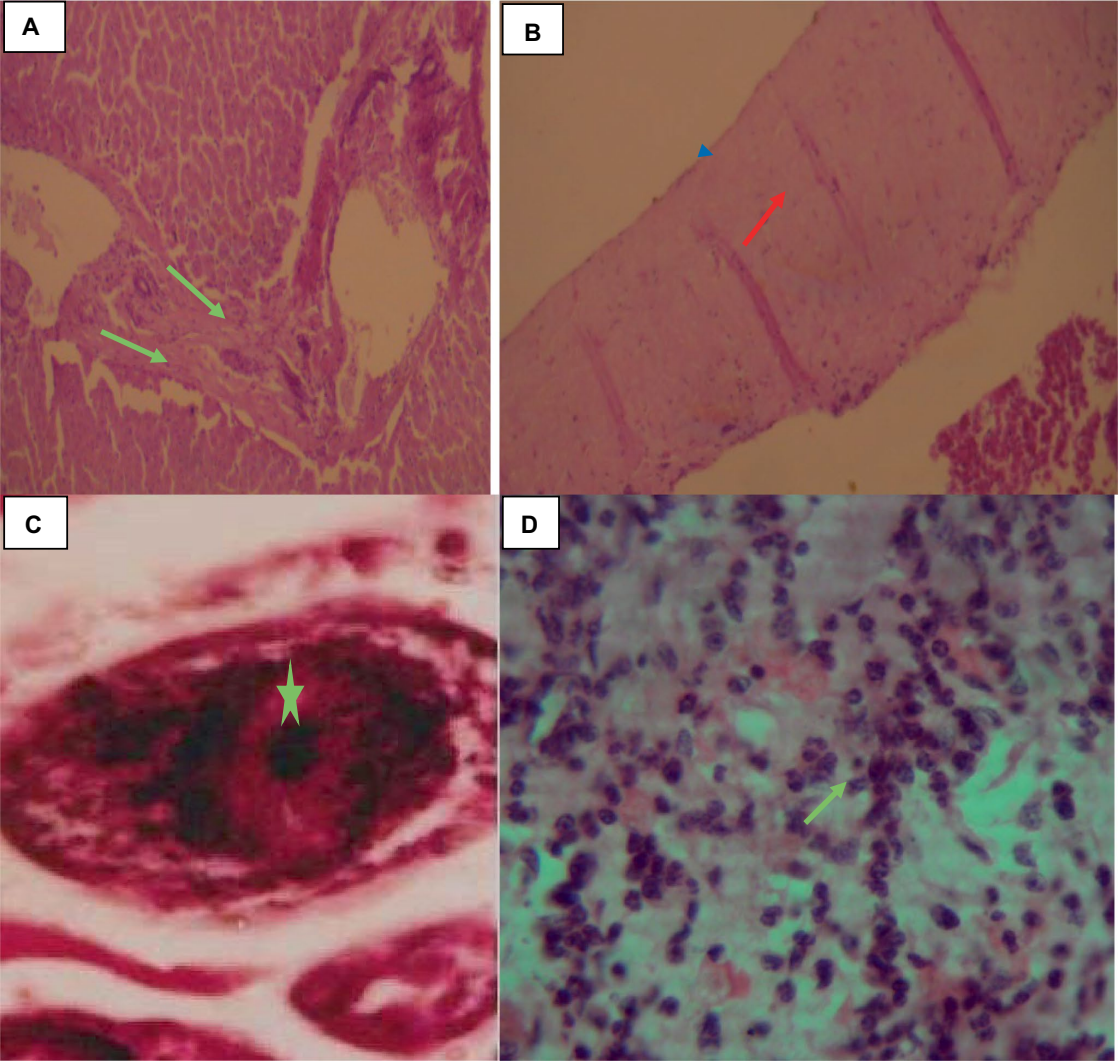
Cattle hepatic hydatidosis 40× magnifications. **(A)** Dilated and fibrotic portal vein with an oncosphere in the portal circulation (light green arrow). **(B)** Middle laminated cyst layer (red arrow) and inner germinative endocyst layer (arrowhead). **(C)** Hydatidosis protoscolices (star). **(D)** Infiltrated macrophages, lymphocytes, and a few eosinophils.

Histologically, the cysts were composed of three layers: the fibrous outer pericyst, the laminated middle ectocyst layer, the germinative inner endocyst layer, and protoscolices, which develop into the daughter cyst. On the oncosphere in the portal circulation, a few parasitic migration tracts could be seen. The liver’s parenchyma was edematous, heavily fibrosized, infiltrated with inflammatory cells, and had deposits of calcified materials around the cyst wall. Higher magnification of the liver parenchyma surrounding the cyst wall revealed eosinophil, lymphocyte, and macrophage infiltration shown in Figure 4.

### Hydatidosis kidney

**Figure 5:**
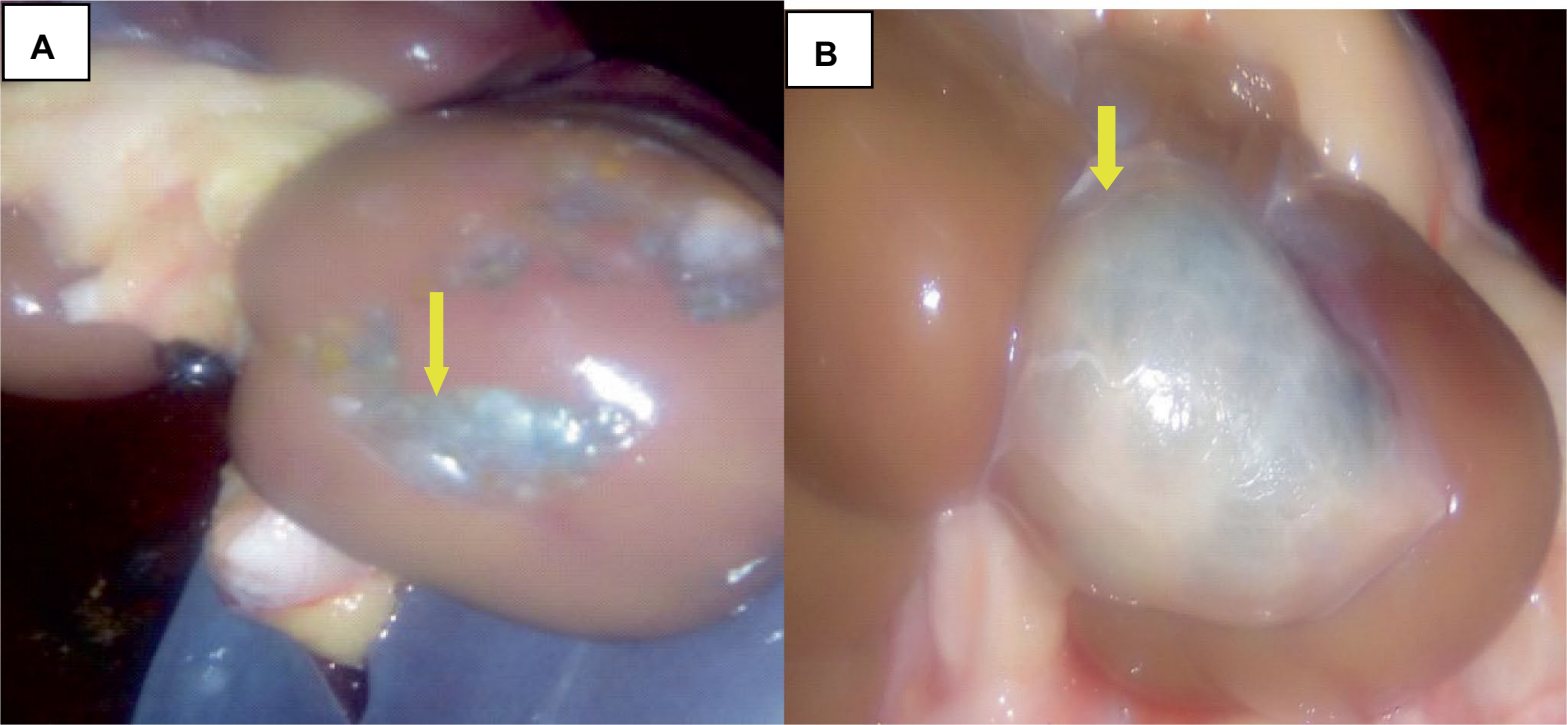
Cattle kidney cysts, macroscopic changes. **(A)** Cysts on the cortex of the kidney, involving the glomerulus, and circular to tubular arranged cysts in the medullary area (yellow arrow). **(B)** Rounded, blackish cyst situated between the surrounding lobules of the kidney, containing brownish or coffee-colored fluids (yellow arrow).

The rate of hydatidosis-related rejection in cystic kidney disease was 0.6% and one kidney has double small size cysts. On the right kidney’s cortex, there were noticeable, dispersed cysts of different sizes. Cysts were seen on the dorsal surfaces of the kidney. There was a thin structure resembling a honeycomb covering the area where the cyst wall joined the kidney. The cyst’s capsule easily detached from the kidney. A single large cyst, spherical, dark brownish, and fitting in between the kidney’s surrounding lobules, was present on the pole of the left kidney. When this cyst was opened, coffee- or brownish-colored fluids were visible. The capsule was denser, whiter, and more linked than the kidney, proving it difficult to separate (Figure 5).

**Figure 6:**
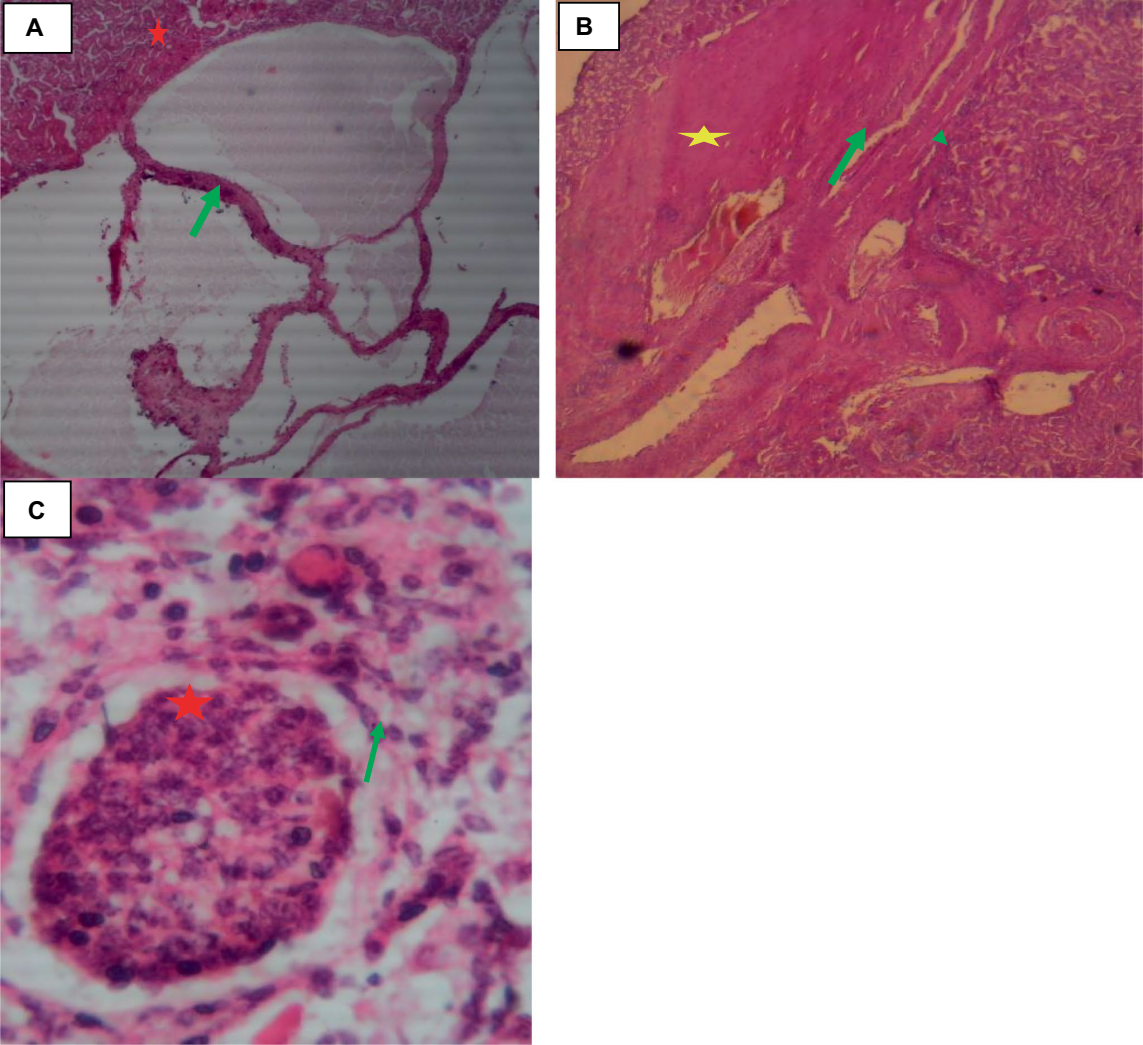
Cattle kidney cysts 40× magnifications. **(A)** Cyst walls lined by cuboidal cells (green arrow), compressed eosinophilic interstitial tissues of the kidney (star), and multiple thin-walled cystic spaces lined with endothelial cells. **(B)** Cyst with an outermost pericyst (arrowhead), a middle ectocyst layer (star), and an inner endocyst (green arrow). **(C)** Necrosis of Bowman’s capsule (green arrow) and fibrotic interstitial tissue with infiltration of inflammatory cells (star).

Histologically, the cortex of the kidney showed multiple sites of cysts that involved the glomerulus, dilating the bowman’s space. A single-cell layer of epithelium coated the cysts. Infiltrating inflammatory cells caused glomerulitis, inflammation, necrosis, and fibrosis of the interstitial tissue as indicated in Figure 6.

### Cysticercosis of liver

**Figure 7:**
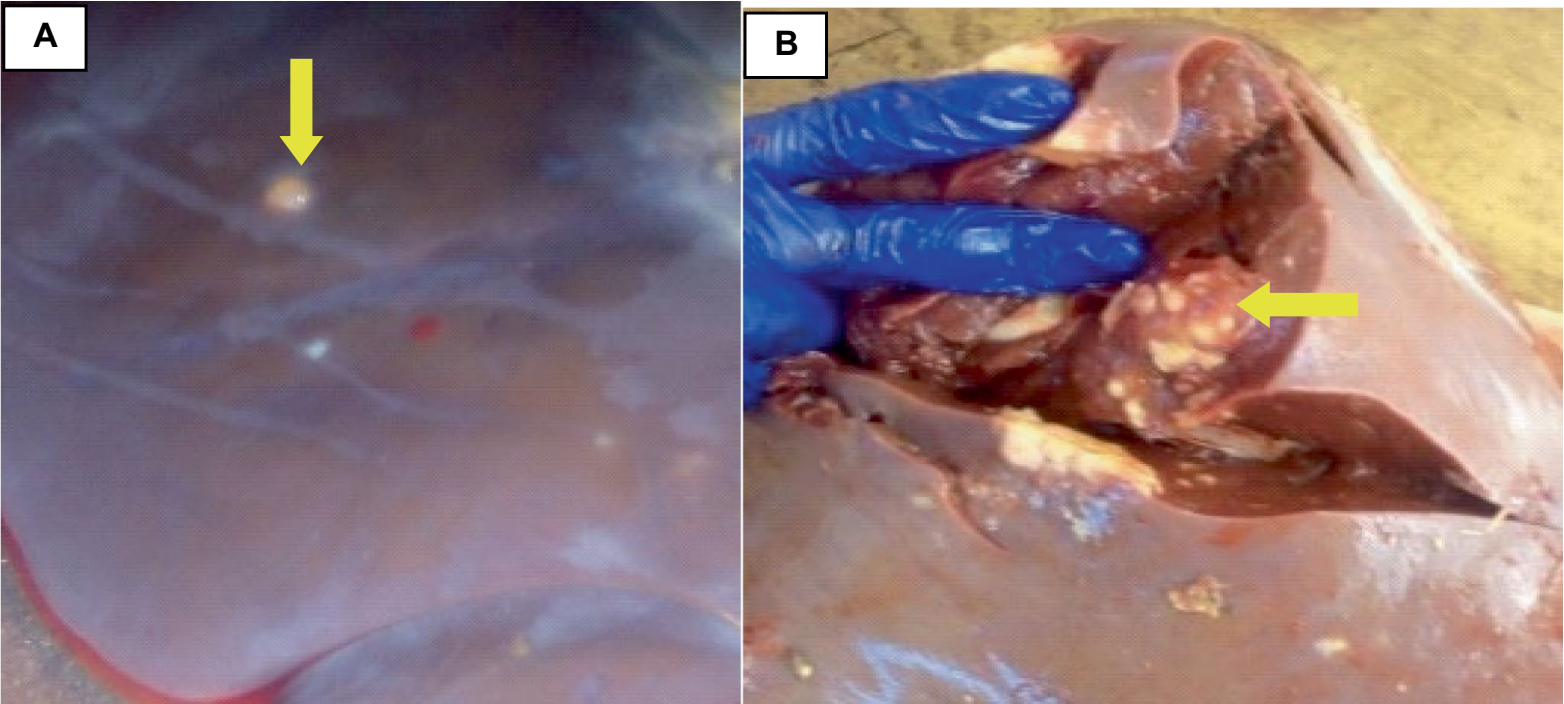
Hepatic cysticercosis in cattle. **(A)** Macroscopic view of the liver showing a somewhat cirrhotic appearance with cysticercosis attached superficially to the liver parenchyma (yellow arrow). **(B)** After incision, the liver appears pale and firm with the presence of cysticercosis (yellow arrow).

The liver was somewhat swollen and enlarged, and a thin film of fibrin with a bloody tint covered the liver capsule. The hepatic capsule was covered in numerous 5–10 mm semi-translucent white cylindrical parasite cysts as indicated in Figure 7.

**Figure 8:**
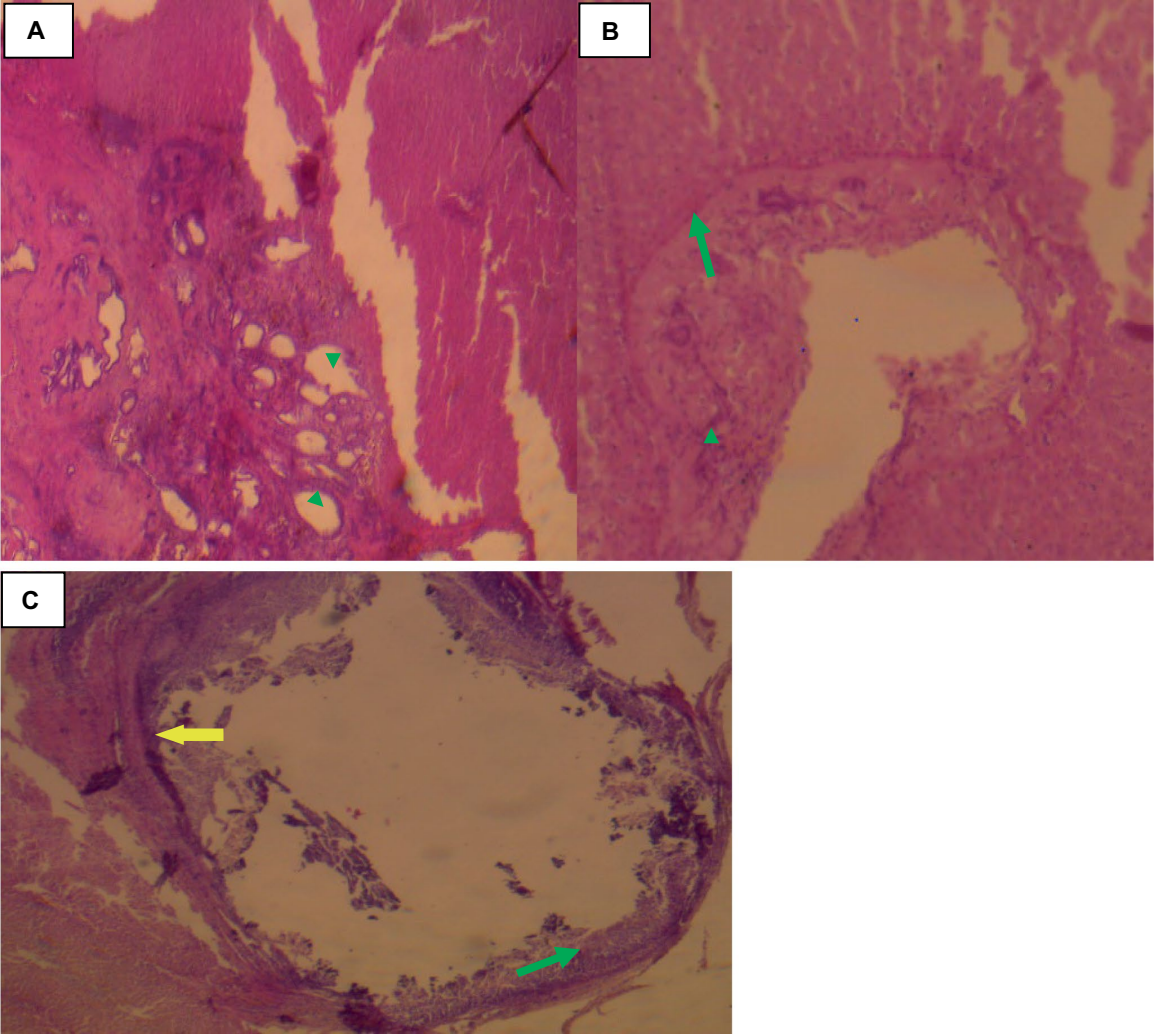
Hepatic cysticercosis in cattle 40× magnifications. **(A)** The hepatic parenchyma is subdivided into islands of irregular hepatocytes, bordered by fibrous connective tissue (arrowhead). **(B)** The cyst wall is formed from two thin eosinophilic membranes (arrowhead) attached to the thick hepatic capsule (green arrow). **(C)** Around the cyst wall, disseminated fibrosis (yellow arrow) and the formation of granulomatous lesions are observed, with infiltration of mononuclear inflammatory cells (green arrow).

Histologically, small quantities of eosinophils, neutrophils, and macrophages were present inside the cavities, together with fibrin, and necrotic cell debris as indicated in Figure 8.

### Cysticercosis tongue

**Figure 9:**
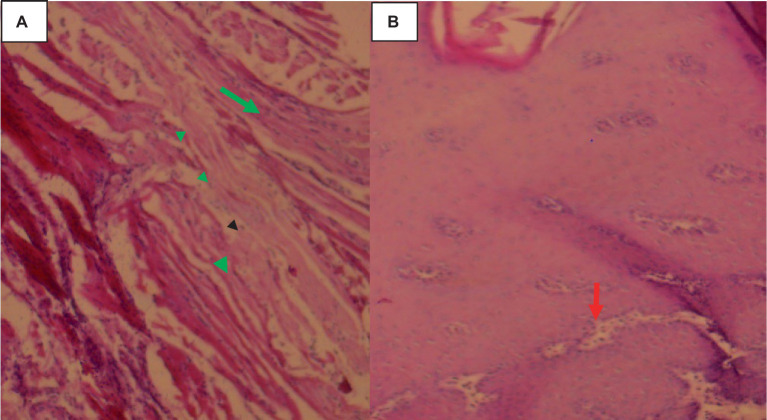
Cysticercosis on the tongue of cattle with 40× magnifications. **(A)** Degenerated and necrotized muscle bundles of the tongue (arrowhead) with infiltration of a few inflammatory cells (green arrow). **(B)** Pale discoloration of the squamous epithelium on the dorsal aspect of the tongue, with distended taste buds and a loss of their round shape (red arrow).

In the tongue muscle, a few intact calcified cysts were detected with Calcified and small size cysts-like structure on lateral aspect tongue. The host reaction was predominantly a proliferation of fibroblast and a relatively thinner collagenous layer. Muscle fiber atrophy, fibroblasts and degeneration were more pronounced as indicated in Figure 9.

## Discussion

Considering meat food safety and public health concerns, meat inspection and food animal health management are very important. Red offal condemnation was primarily caused by zoonotic metacestodes, with unexpected result was found for *C. bovis* and Cystic echinococcosis. The hydatid cysts accounted for 4.12% of lung rejection cases, with liver and kidney accounting for 3 and 0.6% of cases, respectively. This result finding agree with (4) regarding the prevalence of the prevalence and red offals condemnation by *C. bovis* and Cystic echinococcosis of lung 9.6%, liver 3.7%, and kidney 0.6%. The other previous study also showed that (4) lung 7.6%, liver 1.6%, and kidney 0.5% were condemned and unfeet for human consumptions; and (8) reported that lung 6.3% and liver 3.6% infested with cysts and condemned on the meat inspection.

When comparing those results with others previous reports the present research work result reduced in the prevalence report showed some difference (22). This might be due to the sample collection size and animals management system, whether the animal is in intensive or semi-intensive conditions, which could reduce prevalence. Also, the farmers’ deworming practices might be attributed for cysts calcification.

Cysts distribution in different organs of the cattle showed that the lungs 63.03% and liver 33.6%. This because of predilection sites for hydatid cysts are the lung and liver (4, 22) if the animals are infected with *Echinococcus* the cysts developed on those organs and then the liver and lung condemned. This might be justified by the fact that the lungs and liver are the first capillary beds encountered by the migrating *Echinococcus* oncosphere (hexacanth embryo). The embryo first negotiates the liver and lungs’ filtering systems before interacting with any other peripheral organs (4). In addition to host factors, such as the strain of *E. granulosus* involved, cyst location and form are also controlled by parasite factors (4). The occurrence of this difference in the red offal condemned rate by bovine hydatidosis: is most likely due to by the origin and age of the animal, epidemiological risk factors vary from farm to farm and district to district, and animal health preventive measures. All these could have powerful impact on *E. granulosus* diseases transmission and lifecycle of the parasite. There was a statistically significance association between rejection of the lung and kidney by hydatidosis (*p* < 0.05) and animal body condition score.

The meanwhile rejection rate of the liver 0.6% and tongue 0.3% by *C. bovis* and it was not as statically significance when it compared to the rejection rate of the liver and lung by hydatid cysts (23). However, red offal rejection rate due to *C. bovis* and any other risk factors was not statically significance (*p* > 0.05). The reduction of *C. bovis* prevalence and rejection rate of organs by bovine cysticercosis may be due to the awareness of the farmers for animal health and managements, with disease control and eradication initiatives, a clean environment, and reduce raw meat consumption habits. Therefore, the prevalence rate of bovine cysticercosis is likely due to staff awareness and environmental hygiene through proper latrine use, which may contribute to less contamination of grazing land by human excreta containing *T. saginata* eggs, which in turn leads to a reduction of the chance of infection of the intermediate host, cattle (24).

Examining the cysts sizes, it was found that 13.63% were large size, 59.1% were small size, and 27.27% were medium size. Regarding viability and fertility, it was found that 5.95% of the cysts were viable and 11.9% were non-viable, 34.52% were sterile, and 47.62% were calcified. Based on the results that showed that 50.4% of the hydatid cysts from cattle were calcified, 21.01% fertile the current findings agree with Worku (3). The calcification of the cyst is may be happened due to the farmers ‘deworming practice on their cattle with different anti-parasitic drugs. The various parasite strains, the animal’s immune system, and the duration of the infection could all have an impact on the variation in calcification rate, sterility, and fertility (25).

However, the current findings show variations in the fertility rates of the different red offal; the higher fertility rate among lung cysts, this supported by Getaw et al. (26). However, the current research contradicts on the report Gebremeskel et al. (24) in the cattle with hepatic cysts were more fertile than on the pulmonary cysts. This is most likely to be different hydatid cysts strains are found in various climates condition and animal husbandry systems (27).

Pathological changes of red offal, such as edema, portal vein dilation, and hepatocyte atrophy, were evident in the liver where cyst attached wall. Around the cyst in the lung, there is also evidence of atelectasis, bronchiolar collapse, and emphysema. Histologically, three layers of residue and protoscolesis have been seen with microscopic visualizations. Hydatid-infected lungs and livers exhibited considerable pathological abnormalities restricted in and around cyst wall, according to (26, 27) these changes persisted up to a 5 cm cyst.

The lung surface exhibited thickening, atelectatic changes, emphysematous patches, paleness, and a coating of cysts of varying sizes in cases of hydatidosis. Large and small cysts were also discovered deep protrude to lung parenchyma, which led to a significant enlargement. The cysts have clear to turbid fluids color inside the cysts and this gave to the cyst a gray or yellowish appearance and the others had clear to slightly opaque fluid and were mushy. An excessive number of hydatid cysts of various sizes, either fully or partially stuck in the lung parenchyma, were seen in this lung-related finding, especially in the diaphragmatic lobes as described (28, 29).

On the calcified daughter cysts were identified as fibrous (outer pericyst), laminated (middle ectocyst layer), and germinative (inside endocyst layer). The lung parenchymal cyst was fibrotic and covered with calcified debris along with macrophages, lymphocytes, and eosinophil (25). There was bleeding and congestion in the lung parenchyma next to the cysts, which were either atelectatic or emphysematous. The adventitial layer that surrounded the echinococcal cyst had hemorrhagic foci, congestion in the border zone between the adventitia and lung tissue, and a zone of fibrous and connective tissue, according to (25).

The hydatidosis-related cystic liver is characterized by an enormous number of different-sized cysts filled with transparent fluid on the liver’s surfaces. Cysts cavities have different sizes and capsule thicknesses were seen at the incision site. Certain cysts had thin, white to slightly yellowish capsules, while others had thick, fibrous, yellowish capsules with calcified material inside. Particularly in the fully infected liver with cysts, the liver had a pale, hard-to-incise, slightly raised nodular structure. This outcome has been compared to the liver, which is thought to be one of the most preferred locations for the formation of hydatid cysts.

The liver calcifications, blood spots, an inflammatory area surrounding cysts, and pale, blunt liver edges were detected. A few parasitic migratory tracts were observed in the circulation of the portal (22). The liver’s parenchyma under the microscope was infiltrated with inflammatory cells, edematous, extremely fibrotic, and had deposits of calcified materials surrounding the cyst wall. On the microscopic evaluation of liver histopathology showed that the areas close to the cyst wall showed fibrosis, cellular infiltration, congestion, hemorrhage, hepatocyte necrosis, and atrophy (25). The infiltration was mainly seen in the inner side of the fibrous capsule, which appeared broad, as well as the sinusoids and the central.

From the dead cysts nodular, solid, whitish lesions containing a yellowish substance were detected and this result agree with the finding of Ibrahim et al. (30). The cysts were penetrating, composed of lymphoid cells, covered in fibrosis, and characterized by caseous and calcareous materials, multinucleate big cells, and histiocytes in the palisade. Granulation was occasionally visible on the lesion borders. Fibrous nodules with mixed or lymphoid infiltrates were detected.

Visible, dispersed cysts of different sizes were present in the kidney’s surface. On the kidney’s dorsal surfaces, cysts were visible. The area where the kidney and cysts wall attached was covered in a thin structure resembling a honeycomb. On the left kidney’s pole, there was a single, large cysts that was spherical, dark brownish, and fit in between the kidney’s surrounding lobules. The fluid inside these cysts was coffee or brownish-colored when it was opened. This is related to the numerous cysts that were found in the glomerulus; these cysts were spherical, thin-walled, variable in size, and had flattened epithelium along with transparent, watery fluids (31). Histologically speaking, the kidney’s cortex displayed several cyst locations involving the glomerulus, dilation of Bowman’s space, and the presence of primitive glomerular tufts. There was only one layer of epithelial cells covering the cysts.

On red offals of the tongue calcified cysts were detected. The squamous epithelium of the tongue was darkened. The inflammatory cells infiltrated and fibroblasts proliferated as the primary components of the host response. Muscle fibers of the tongue organ showed significant degenerative alterations and a central necrotic core on histological inspection, and that cysts of various sizes were retrieved, some of which were calcified this supported by the finding Canda et al. (29) and Costa et al. (31). The nuclei were moved to the flanks and there was extensive damage of tongue cells where the cyst attached.

## Conclusion

Edible organs rejected during the meat inspection procedure due to the presence of zoonotic metacestode diseases. The primary causes of organ rejection at the abattoir were zoonotic metacestode diseases, including hydatidosis and cysticercosis, which may also be prevalent in other slaughterhouses in Ethiopia. Hydatidosis was more frequently responsible for the rejection of red offal compared to cysticercosis. Abnormal changes were observed in all organ tissues surrounding the cyst wall. A postmortem diagnosis of cysticercosis and hydatidosis can be achieved through gross and histological tests. Considering the common detection of zoonotic metacestode cysts in red offal, public health concerns should be addressed. Cattle should be strategically dewormed with appropriate anthelmintics at the right time, and measures should be taken to control stray dogs. Additionally, raw meat consumption should be avoided to disrupt the life cycle of cestodes.

## Data availability statement

The original contributions presented in the study are included in the article/supplementary material, further inquiries can be directed to the corresponding author.

## Ethics statement

The animal study was approved by Institutional Ethical Review Board (IRB) of college of Veterinary Medicine and Animal Sciences, University of Gondar, Ethiopia. It has given at reference (Reference No: CVMAS.Sc.16.282025). The study was conducted in accordance with the local legislation and institutional requirements.

## Author contributions

DA: Formal analysis, Methodology, Writing – original draft, Writing – review & editing. MM: Formal analysis, Methodology, Writing – review & editing. YD: Conceptualization, Data curation, Writing – review & editing. AM: Methodology, Supervision, Writing – original draft, Writing – review & editing.
